# Chromium Selectively Accumulates in the Rat Hippocampus after 90 Days of Exposure to Cr(VI) in Drinking Water and Induces Age- and Sex-Dependent Metal Dyshomeostasis

**DOI:** 10.3390/toxics12100722

**Published:** 2024-10-03

**Authors:** Samuel T. Vielee, William J. Buchanan, Spencer H. Roof, Rehan Kahloon, Elizabeth Evans, Jessica Isibor, Maitri Patel, Idoia Meaza, Haiyan Lu, Aggie R. Williams, J. Calvin Kouokam, Sandra S. Wise, Luping Guo, Rachel M. Wise, Jamie L. Wise, Lu Cai, Jun Cai, John P. Wise

**Affiliations:** 1 Pediatrics Research Institute, Department of Pediatrics, University of Louisville, Louisville, KY 40292, USA; samuel.vielee@louisville.edu (S.T.V.); spencer.roof@louisville.edu (S.H.R.); rehan.kahloon@louisville.edu (R.K.); lu.cai@louisville.edu (L.C.);; 2 Department of Pharmacology and Toxicology, University of Louisville, Louisville, KY 40292, USA; idoia.isusi@louisville.edu (I.M.); sandra.wise@louisville.edu (S.S.W.);; 3 Department of Pharmaceutical Sciences, College of Pharmacy, University of New Mexico, Albuquerque, NM 87131, USA

**Keywords:** hexavalent chromium, neurotoxicity, essential metals dyshomeostasis, age differences, sex differences

## Abstract

Hexavalent chromium (Cr[VI]) is a widespread environmental pollutant in air and water that is primarily attributed to industrial pollution. The current maximum contaminant levels (MCLs) for drinking water from the World Health Organization and the U.S. Environmental Protection Agency (0.05 and 0.1 mg/L, respectively) were set based on contact dermatitis and warrant further toxicological investigation. While Cr(VI) is neurotoxic and accumulates in the brain, most animal studies only report whole-brain Cr, leaving large knowledge gaps. Few studies consider differences between ages or sexes, and fewer consider essential metal dyshomeostasis. We sought to investigate where Cr accumulates in the brain, considering sex and age differences, following a 90-day drinking water exposure to current MCLs. Here, we report Cr levels in six brain regions of rats exposed to drinking water Cr(VI). We observed Cr only accumulated in the hippocampus, and only in older females. We further assessed changes to essential metals in the hippocampus, observing opposite effects across sexes and between young rats compared to older rats. In sum, our data indicate drinking water Cr(VI) selectively targeted the hippocampus, with geriatric females accumulating the most Cr, and induced significant essential metal dyshomeostasis even in tissues lacking evident Cr accumulation.

## 1. Introduction

Environmental pollution is a persistent and ubiquitous threat to human health, with environmental pollutants inducing negative health effects in humans across all demographics [1]. Metals are a particularly egregious form of environmental pollution, as they are naturally occurring, often toxic at low levels, and remediation is difficult since most metals cannot be metabolized or degraded to less toxic forms. Exposure to metal pollution is linked to a wide variety of human diseases, impacting all organ systems [2,3,4]. Neurotoxicity is well described for many metals (e.g., Pb, Mn, Hg), but many knowledge gaps still exist for chromium [5,6,7,8,9,10,11,12,13,14,15].

Chromium (Cr) is a naturally occurring metal highly desired in industry for its bright colors, anticorrosive properties, and hardness. New applications for Cr are developing for green energy, and this new application will increase the demand with a market-projected $6 billion profit by 2031 [16,17,18]. Chromium is most often found in two valence states: trivalent chromium (Cr[III]) is innocuous, while hexavalent chromium (Cr[VI]) is highly toxic and a known human lung carcinogen [19]. Further, Cr(VI) is ranked 17th on the Substance Priority List from the Agency for Toxic Substances and Disease Registry (ATSDR), along with four other neurotoxic metals (i.e., lead, arsenic, mercury, cadmium) [19]. Environmental pollution of Cr(VI) is primarily attributed to industrial sources, though some rare geogenic sources can also contribute to high drinking water Cr(VI). The current maximum contaminant level (MCL) for total Cr in drinking water is 0.05 mg/L according to the World Health Organization (WHO) and 0.1 mg/L according to the U.S. Environmental Protection Agency (U.S. EPA). Most studies report city drinking water Cr(VI) levels below these limits, though several reports include drinking water levels approaching or exceeding the WHO and U.S. EPA maximum contaminant levels [20,21,22,23,24]. The highest Cr(VI) level reported in U.S. drinking water was from a well in Midland, Texas reported at 5.41 mg Cr(VI)/L, though the source was not identified [25]. The highest Cr(VI) level reported globally was 20 mg/L following a chemical spill incident in Jinzhou, Liaoning, China that started in 1961 [26]. Geogenic sources also contribute to Cr(VI) drinking water contamination, with sites identified in California, North Carolina, and Greece [18,19,20]. One group identified the geogenic source as intrusive and metamorphic meta-igneous bedrock leaching Cr(VI) into well water and groundwater in North Carolina, USA, with some sources exceeding the state advisory level of 0.00007 mg/L [23,24]. Critically, this group also reported a strong correlation between total Cr levels and Cr(VI) levels in groundwater, indicating the majority of total Cr signal was from Cr(VI) [24].

Cr(VI) toxicity is well described in peripheral organs and is best characterized as a potent lung carcinogen [27]. Neurotoxicity remains an under-recognized aspect of Cr(VI) toxicity. Human studies report Cr(VI) exposure linked to autism spectrum disorder, olfactory dysfunction, polyneuropathy, acute schizophrenia, and motor neuron disease [28,29,30,31,32,33,34]. The reported effects of Cr(VI) on rodent behavior primarily considered motor activity or locomotor function following unrealistically high Cr(VI) levels, though we recently reported low concentrations of Cr(VI) in drinking water (0.05 or 0.1 mg/L) induced significant behavioral changes in grip strength, spatial memory, social memory, exploration tendency, and rearing behaviors within 90 days of exposure [35,36,37,38,39,40].

The field remains sparse on details for where Cr accumulates in the brain. Human data are limited to postmortem reports without known Cr(VI) exposures, but collectively suggest Cr accumulates in the brain with age and the greatest accumulation was reported in the temporal cortex and pituitary gland [28]. Two rodent studies assessed regional Cr accumulation in rats after 100 or 500 mg/L drinking water exposure to potassium dichromate for 30 days, though these studies only assessed Cr accumulation in the hypothalamus and pituitary gland [41,42]. A recent mouse study reported Cr(VI) crossed the blood–brain barrier of 6- to 8-week-old (young/adolescent) male mice after 14 or 28 days intraperitoneal exposure to 6 mg/kg/day of potassium dichromate [43]. The authors reported significant increases across ten brain regions (olfactory bulb, prefrontal cortex, somatosensory cortex, striatum, hippocampus, thalamus, hypothalamus, cerebellum, midbrain, medulla), with the largest accumulation in the hypothalamus [43]. Understanding regional accumulation of Cr(VI) is essential to fully comprehend Cr(VI) neurotoxicity and the associated risk of neurological diseases/disorders.

Essential metal homeostasis (Fe, Cu, Se, Mg, Co, Mn, Zn) is critical to brain function and deviation from normal levels results in neurological dysfunction, with several metals exhibiting narrow margins of homeostasis [44,45,46,47]. For example, Cu deficiency results in Menke’s disease while excess Cu results in Wilson’s disease [44]. We have little understanding for how Cr affects essential metal homeostasis in the brain. One study reported decreased levels of Fe, Cu, and Zn, but increased Mn, in the mouse brain after a single intraperitoneal injection of 8 mg/kg Cr(VI), but this study did not consider region-specific effects and only assessed effects in young males [48]. 

Here, we sought to determine where Cr accumulates in the brain following environmentally relevant exposures in drinking water, considering differences across sexes, ages, and six brain regions (hippocampus, cortex, hypothalamus, cerebellum, striatum, brainstem). We further considered the effects of Cr(VI) drinking water exposure on levels of essential metals (Fe, Cu, Se, Mg, Co, Mn, Zn) in the hippocampus, considering sex and age differences in our model. To address the effect of Cr(VI) on brain metals of rats at different ages, we employed a “Toxic Aging Coin” approach [49]. Briefly, the *heads* side considers how an individual’s age influences chemical toxicity, while *tails* considers how chemicals accelerate biological aging (i.e., act as gerontogens) [50,51]. To employ this approach, we exposed male and female Sprague-Dawley rats from three different postnatal ages (3-, 7-, or 18-months-old) to 0, 0.05, or 0.1 mg Cr(VI)/L drinking water for 90 days. After exposure, we microdissected brains to isolate the brainstem, cerebellum, cortex, hippocampus, hypothalamus, and striatum for metallomic analyses using inductively couple plasma-mass spectrometry (ICP-MS).

## 2. Methods

### 2.1. Animals and Drinking Water Exposure

**Figure 1:**
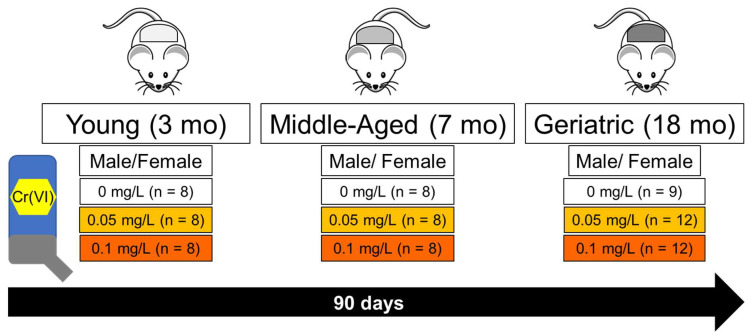
**Rat model to assess Cr(VI) neurotoxicity using the Toxic Aging Coin approach.** We enrolled 162 Sprague-Dawley rats (both sexes) of three different ages (3-, 7-, and 18-months-old), which were exposed to 0, 0.05, or 0.1 mg Cr(VI)/L in drinking water for 90 days (~13 weeks). At the end of exposure, rats were sacrificed and brains were microdissected to isolate the brainstem, cerebellum, cortex, hippocampus, hypothalamus, and striatum. Tissue was digested and analyzed for metal content using ICP-MS.

Rats were maintained in facilities fully accredited by AAALC International, and all experiments were performed under protocol IACUC 21934, approved by the University of Louisville Institutional Animal Care and Use Committee in accordance with the ARRIVE guidelines and the National Institutes of Health Guide for the Care and Use of Laboratory Animals. The study design is shown in Figure 1. Data presented here included 162 male and female Sprague-Dawley rats purchased from Envigo (Indianapolis, IN, USA), enrolled in the study at 3-, 7-, or 18-months old. Upon termination of the study, groups were 6-, 10-, and 21-months old, respectively. Rats were fed ad libitum with a rodent diet with 10% kcal from fat (Research Diets, D12450Ji). Rats from each age group were divided into 3 exposure groups, for a total of 9 groups per sex: study groups received tap water, 0.05 mg Cr(VI)/L in tap water, or 0.1 mg Cr(VI)/L in tap water. Critically, these drinking water levels reflect the MCLs set by the WHO and U.S. EPA, respectively. Young and middle-aged groups included 8 rats each, while geriatric groups included 9 or 12 rats for control and Cr(VI)-treated groups, respectively, to account for attrition due to age. The sample size (*n* = 8) per group enables us to detect an effect size (i.e., the ratio of group mean difference over within-group standard deviation) of 1.8 with a power of 80% at a significance level of 0.05.

Cr(VI) was administered as sodium chromate (Sigma Aldrich, St. Louis, MO, USA, 307831) dissolved in tap water. Cr(VI) exposures were prepared weekly from 1000× stocks in tap water: 50 mg Cr(VI)/L and 100 mg Cr(VI)/L stocks were diluted to 0.05 and 0.1 mg Cr(VI)/L final concentrations in tap water. Body mass and drinking water mass were measured weekly. For QA/QC, drinking water samples were collected weekly for metals analyses to validate Cr(VI) concentration (Figure S1). Seven rats were lost to attrition during this study. Three animals were found deceased and tissues could not be collected; a geriatric female and 2 geriatric males. Two geriatric control males and one geriatric female (control) were sacrificed during the 10th week of exposure and an additional geriatric female (0.05 mg/L) was sacrificed during the 11th week of exposure due to health complications. We included tissues from these rats in analyses. 

After 90 days exposure, rats were anesthetized via intraperitoneal injection of 100 mg/mL xylazine and 100 mg/mL ketamine (combined at a 1:9 ratio) at a dose of 0.1 mL/kg bodyweight. Rats were euthanized via exsanguination, using cardiac perfusion with 250–300 mL of cold 1× DPBS (without calcium or magnesium) (Corning, New York, NY, USA, 20-031-CV) vacuum-filtered through a 0.22-μm membrane. Rats were decapitated following perfusion. Brains were extracted, rinsed in ice-cold 1× DPBS, and blotted to remove excess liquid. Brains were bisected using a Rodent Brain Matrix (ASI Instruments, Warren, MI, USA, RBM-4000C). One hemisphere was microdissected to isolate the brainstem, cortex, cerebellum, hippocampus, hypothalamus, and striatum. Whole brain masses and microdissected brain region masses were recorded. The cortex was bisected further into the rostral and caudal cortex, with rostral cortex metal levels reported here.

### 2.2. Inductively Coupled Plasma-Mass Spectrometry (ICP-MS) Analysis 

For ICP-MS analyses, all tissues were digested in 70% nitric acid for 3 h at 85 °C. For liquid samples, 100 μL of each sample was digested in nitric acid for 3 h at 85 °C. After acid digestion, samples were cooled to room temperature and further digested with 100 μL 3% hydrogen peroxide for 1 h at room temperature. Following hydrogen peroxide digestion, samples were diluted with Millipore water to a final concentration of 5% nitric acid and filtered through Acrodisc 32-mm, 0.45-μm Supor^®^ filters (4654, Pall Corporation, Port Washington, NY, USA) into trace element-free 15 mL centrifuge tubes (89049-170, VWR Avantor, Radnor, PA, USA). Filtered digestates were stored at −20 °C until ICP-MS analyses. Samples were run alongside reference standard for trace elements in water (NIST1643F, Millipore Sigma, Burlington, MA, USA). Samples were analyzed on an Agilent 7900 ICP-MS in the Integrative Molecular Analysis Core (IMAC) at the University of New Mexico and in the Metallomics Core Facility at the University of Louisville. Initial calibration blank (ICB) and initial calibration verification (ICV) standards were run at the start of the analysis, after every 10 samples, and at the end of the analysis. An internal standard was included in all sample runs to account for any matrix effects. Any samples below the detection limit were reported as ½ the limit of detection.

### 2.3. Statistical Analyses

The sample size (*n* = 8) per group enables us to detect an effect size (i.e., the ratio of group mean difference over within-group standard deviation) of 1.8 with 80% power at a significance level of 0.05. Outliers were removed from analyses if the data point was 10× greater than the average of the remaining group. Normality was assessed using the Anderson-Darling test (α = 0.05). Statistical differences were assessed using a Student’s *t*-test with a Welch’s correction or a Mann-Whitney U test for parametric and non-parametric data, respectively (α = 0.05). Criterion for significance in all tests was *p* < 0.05, though values of *p* < 0.1 were noted. All statistical analyses were conducted using GraphPad Prism 9 (v.9.5.1). Data are expressed as mean ± standard error of the mean (SEM).

## 3. Results

### 3.1. Cr Brain Accumulation Is Region-, Sex-, and Age-Specific

**Figure 2:**
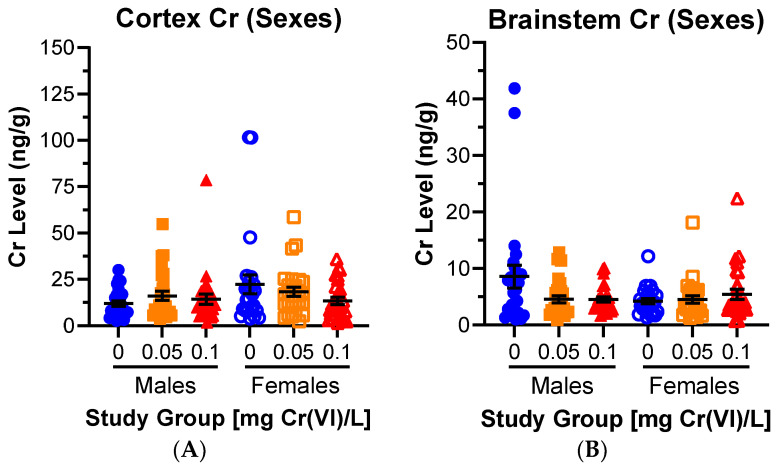

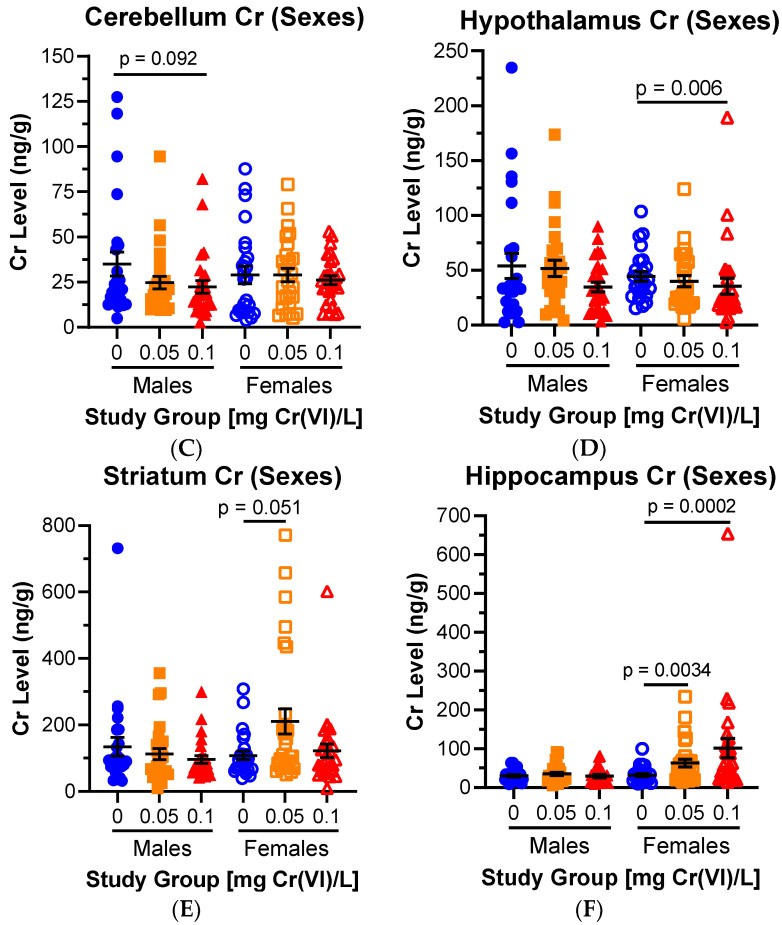
**Cr selectively accumulated in the female hippocampus following drinking water exposure.** Regional Cr data were grouped by sex but not age. (**A**–**D**) ICP-MS analyses revealed Cr did not accumulate in the cerebellum, cortex, brainstem, or hypothalamus. (**E**,**F**) Cr accumulated in the female striatum after exposure to 0.05 mg/L and in the female hippocampus after exposure to both Cr(VI) concentrations. Data represent mean ± SEM.

We isolated six brain regions to assess Cr accumulation using ICP-MS. We observed the highest Cr levels in the hippocampus, hypothalamus, brainstem, and striatum; and the lowest levels in the rostral cortex. We first considered results as Cr(VI) effects on a population by pooling all ages and sexes across exposure groups, and only observed a statistically significant effect for the hippocampus after exposure to 0.05 mg/L (*p* = 0.0242). We then separated these population comparisons by sex (Figure 2). Comparing across sexes, independent of age, we observed no Cr accumulation in the cortex or brainstem of either sex (Figure 2A,B). Unexpectedly, exposure to 0.1 mg/L decreased Cr levels in the male cerebellum and female hypothalamus (*p* = 0.092 and 0.006, respectively) (Figure 2C,D). Several outliers were removed as they were an order of magnitude greater than the mean of the rest of the group, including: two hippocampal values (310.0 ng/g from a young female 0.1 mg/L; 946.9 ng/g from a geriatric male 0.05 mg/L); one striatal value (1297.9 ng/g from a geriatric male 0.1 mg/L); one hypothalamic value (3146.6 ng/g from a middle-aged female 0.05 mg/L); and two brainstem values (71.8 ng/g from a geritatric male 0.1 mg/L; and 1086.6 ng/g from a geriatric female 0.05 mg/L). Removing these outliers did not change interpretation of the data. 

Cr accumulated in the striatum of females exposed to 0.05 mg/L (*p* = 0.051), but we did not observe any changes in striatal Cr levels for other groups (Figure 2E). Cr significantly accumulated in the female hippocampus after exposure to both concentrations of Cr(VI), but this effect was absent in males: 31.60 ± 4.15, 63.03 ± 10.08, and 101.5 ± 24.93 ng Cr/g tissue for control, 0.05, and 0.1 mg/L exposed females, respectively (*p* = 0.0034 and 0.0002 for 0.05 and 0.1 mg/L exposed rats, respectively) (Figure 2F).

**Figure 3:**
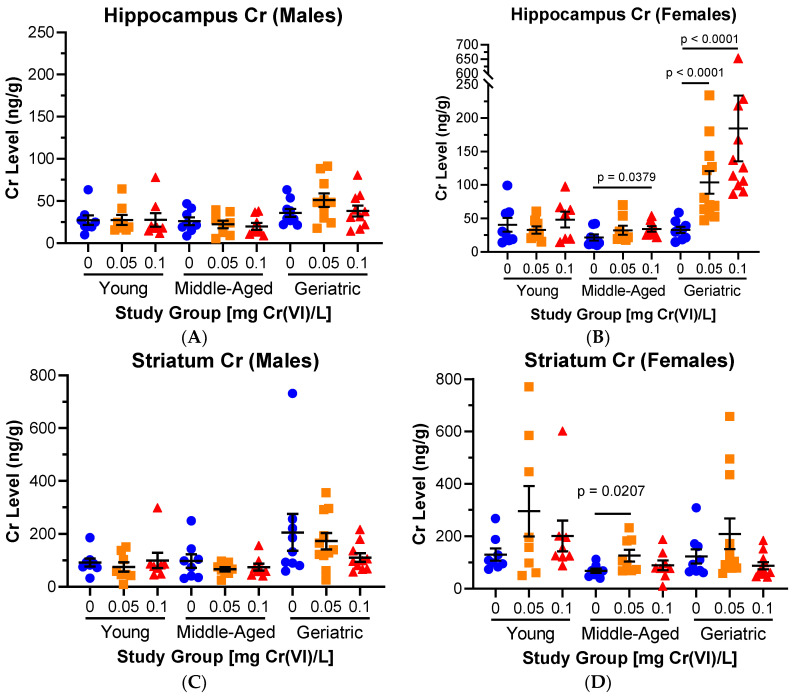
**Hippocampal Cr accumulation was significantly elevated in middle-aged and geriatric females.** Regional Cr data were grouped by age and sex. (**A**) Hippocampal Cr levels were largely unaffected in male rats, regardless of age. (**B**) In females, Cr significantly accumulated in a concentration-associated manner in the middle-aged and geriatric hippocampus. (**C**) Striatal Cr levels were largely unaffected in male rats, regardless of age. (**D**) In females, Cr significantly accumulated in middle-aged striatum after exposure to 0.05 mg/L. Data represent mean ± SEM.

We next compared results across age groups by sex, to consider our results in the context of the *heads* side of the Toxic Aging Coin. For both sexes, Cr levels remained unchanged, regardless of age, in the cerebellum, cortex, hypothalamus, and brainstem. Male hippocampal and striatal Cr levels were largely unchanged, regardless of age (Figure 3A,C). Female hippocampal Cr levels exhibited notable age differences: young female hippocampal Cr levels were unaffected, but middle-aged females exhibited a concentration-associated increase in hippocampal Cr which was significant after exposure to 0.1 mg/L (21.38, 23.27, and 34.31 ng Cr/g tissue for control, 0.05, and 0.1 mg/L exposed females, respectively; *p* = 0.1049, 0.0379). Geriatric females exhibited the largest hippocampal Cr accumulation, with 3.2× and 4.2× higher Cr in exposed groups (32.83 ± 10.41, 103.7 ± 16.93, and 137.5 ± 16.22 ng Cr/g tissue for control, 0.05, and 0.1 mg/L exposed females, respectively; *p* = 0.0003, 0.0002) (Figure 3B). Female striatal Cr levels exhibited a consistent pattern across ages, with Cr accumulation in the 0.05 mg/L groups, which was statistically significant in middle-aged females (*p* = 0.0207; Figure 3D).

### 3.2. Cr(VI) Induced Hippocampal Metal Dyshomeostasis with Major Age and Sex Differences

**Figure 4:**
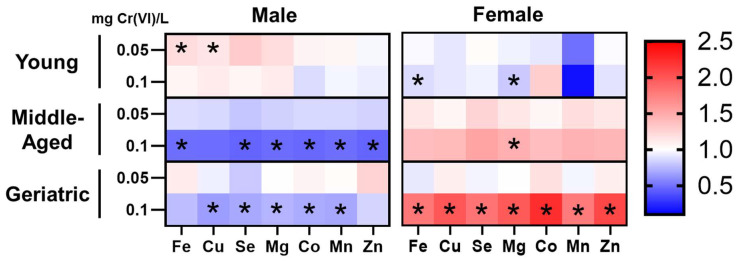
Cr(VI) induced age- and sex-specific metal dyshomeostasis in the hippocampus. Cr(VI) in-creased Fe and Cu levels in young males but decreased Fe and Mg levels in young females. Cr(VI) decreased all essential metal levels in the middle-aged and geriatric male hippocampus, but increased levels in middle-aged and geriatric females. Red color indicates elevated metal levels; blue color indicates decreased metal levels * *p* < 0.05.

We next considered how Cr(VI) impacts essential metal levels in the hippocampus. We assessed hippocampal levels of Fe, Cu, Se, Mg, Co, Mn, and Zn, and reported the fold-change relative to age-matched controls in Figure 4. Raw data for essential element levels can be found in Table 1. We observed opposite effects between sexes at all ages, and opposite effects between young rats and older rats within the same sex.

In young rats, we observed most essential metals were elevated in males but decreased in females. Specifically, Cr(VI) significantly increased hippocampal Fe and Cu levels in young males exposed to 0.05 mg/L; whereas Fe and Mg were significantly reduced in female rats exposed to 0.1 mg/L.

Contrasting young males, we observed Cr(VI) predominantly reduced levels of essential metals in middle-aged and geriatric males; effects were significant for all essential metals (except Cu) in middle-aged rats exposed to 0.1 mg/L, and all but Fe and Zn in geriatric rats exposed to 0.1 mg/L. Contrasting young females and age-matched males, essential metals were predominantly elevated in middle-aged and geriatric females. Notably, exposure to 0.1 mg/L significantly increased Mg levels in middle-aged females and significantly increased all essential metal levels in geriatric females.

## 4. Discussion

Human exposure to Cr(VI) can contribute to motor neuron disease, autism spectrum disorder, olfactory dysfunction, acute schizophrenia, and likely other neurological conditions [28]. Studies linking Cr(VI) exposures are in part limited due to a critical knowledge gap regarding where Cr accumulates in the brain. Compiling data from studies reporting human brain Cr levels from non-occupationally exposed people, the highest levels were observed in the temporal cortex and pituitary [28].

Reports from animal studies predominantly report whole brain Cr levels, and only three other studies reported brain Cr levels after drinking water exposures in rodents. One study reported a whole-brain Cr level of 0.50 ug/g (500 ng/g) after 1–3 days of orally administered 25 mg/kg potassium dichromate in drinking water [40]. Two other groups have reported on specific brain regions. One group reported Cr levels in the hypothalamus and pituitary of male Wistar rats following 30 days exposure to 100 or 500 ppm potassium dichromate in drinking water; pituitary levels were 274 and 640 ng/g, while hypothalamus levels were 26 and 59 ng/g for 100 and 500 ppm, respectively [41,42]. Another group recently reported regional Cr deposition in ten brain regions (olfactory bulb, prefrontal cortex, somatosensory cortex, striatum, hippocampus, thalamus, hypothalamus, cerebellum, midbrain, medulla) of male C57BL/6J mice following 14- or 28-day i.p. injections of potassium dichromate at 6 mg/kg daily (100 mg/L bolus), and determined the hypothalamus accumulated the highest levels reaching 120.9 ppm (or 120,900 ng/g for comparison) [43]. The highest level they observed in the hippocampus was approximately 40 ppm (or 40,000 ng/g). By comparison, we exposed Sprague-Dawley rats (both sexes) from three distinct ages (3-, 7-, and 18-months old when enrolled) to 0.05 and 0.1 mg/L sodium chromate in drinking water. We observed the highest Cr level (770.9 ng/g) in the striatum of young females (6 months old at sacrifice) exposed to 0.05 mg/L, though there was a lack of apparent accumulation in the 0.1mg/L group. Interestingly, the hippocampus exhibited the most striking pattern of accumulation with a concentration-associated increase in middle-aged and geriatric females, though no apparent accumulation in males or young females. Our data demonstrate Cr brain accumulation is age-, sex-, and region-specific in rats following a drinking water Cr(VI) exposure. We report a 90-day exposure to low concentrations of Cr(VI) in drinking water (0.05 or 0.1 mg/L) led to a selective accumulation of Cr in the rat hippocampus, and these effects were most notable in geriatric female rats. Studies using other exposure routes and animal models also failed to account for age, sex, and brain region in their analyses [39,48,52,53,54,55]. Our data suggest these three factors are critical considerations for the impact of Cr(VI) on brain health.

Our data also indicate Cr(VI) induced essential metal dyshomeostasis in the hippocampus, with opposite effects between sexes and ages (young vs. older), regardless of evident Cr accumulation. Young males exhibited increased essential metal levels after Cr(VI) exposure, whereas middle-aged and geriatric males exhibited significantly decreased essential metal levels. In contrast, young females exhibited decreased essential metal levels, while middle-aged and geriatric females exhibited significantly increased levels. Two studies have reported Cr(VI)-induced metals dyshomeostasis in the brain [48,56]. Doker et al. exposed 6–7-week-old (young/adolescent) male Swiss albino mice to a single dose of 8.0 mg potassium dichromate/kg via intraperitoneal injection [48]. They reported Cr(VI) increased Mn levels in the brain, but decreased Cu, Fe, and Zn levels—effects supported by data in our young rats [48]. Zhu et al. exposed chickens to 22.14 mg potassium dichromate/kg bodyweight by daily oral gavage for 14, 28, and 42 days [56]. The authors reported Cr(VI) decreased Ca, Fe, and Zn levels after 42 days of exposure, though Mn levels were increased [56]. We observed similar effects with Fe and Zn in our middle-aged and geriatric males, though we observed increasing concentrations of these metals in the middle-aged and geriatric female hippocampus. For most metals reported by Zhu et al., brain levels were generally increased after a 28-day exposure despite being decreased after 42 days [56]. Altogether, these data suggest Cr(VI) exposure induces brain metal dyshomeostasis, regardless of age, sex, exposure route, or species—a crucial consideration for relevance to human diseases.

While Cr(VI)-induced changes in accumulation and metal dyshomeostasis are evident and distinct across age and sex, we also noted natural changes in hippocampal essential metal levels from control groups across ages. For example, unexposed males exhibited age-associated increases in Fe, Cu, Se, Mn, Co, and Mg levels, whereas unexposed females exhibited age-associated decreases in Fe, Mn, and Mg levels. If we consider our Cr(VI) effects on metal homeostasis in the context of these natural aging effects, we observe a gerontogenic effect of Cr(VI) on essential metal dyshomeostasis in young rats: young males exhibited elevated essential metals and young females exhibited decreased essential metals in similar patterns to the natural aging effect for each sex. Hence, future assessments of Cr(VI) neurotoxicity should also incorporate a Toxic Aging Coin approach to account for changes in chemical toxicity with age [49].

Brain essential metal dyshomeostasis is reported during brain aging and as a factor in a wide variety of neurological conditions [47,57,58,59,60,61,62]. For example, Fe accumulation is reported for Alzheimer’s disease, Parkinson’s disease, ferritinopathies, Creutzfeldt-Jakob Disease, and various mitochondrial pathologies [47,63,64,65]. Evidence suggests Fe deficiency is associated with cognitive effects, behavioral effects, restless leg syndrome, and degeneration of the CA1 and CA2 regions of the hippocampus [46]. Cu accumulation is associated with amyotrophic lateral sclerosis, Alzheimer’s, Parkinson’s, and Wilson’s diseases; whereas Cu deficiency impairs mitochondrial function and is a phenotype of Menke’s disease [44,47]. Similar links to human diseases (especially Alzheimer’s disease) are reported for Se, Mn, Zn, and Co—emphasizing how detrimental Cr(VI)-induced metal dyshomeostasis may be to human populations [66,67,68,69].

Key factors to consider in Cr(VI) toxicity and essential metal dyshomeostasis include sex, age, duration of exposure, exposure route, and mechanisms of reduction to Cr(III). Studies in human populations reported the highest brain Cr levels in older populations, but we have a limited understanding of the influence of sex and exposure routes in these studies [28]. A lack of a detailed exposure impairs our ability to determine if Cr accumulated in the human brain over a lifetime of exposure or if older individuals are simply more vulnerable to Cr accumulation—as shown in our rat study.

Exposure route is a key consideration for Cr(VI), clearly demonstrated by the fact that Cr(VI) is a known carcinogen via inhalation exposure, whereas it is not via ingestion [19]. Rodent studies considering different exposure routes also demonstrate exposure route can change the brain regions with the highest accumulation; our drinking water study resulted in the hippocampus exhibiting the highest accumulation, whereas a daily i.p. injection resulted in the hypothalamus exhibiting the highest accumulation [43]. From an ingestion exposure as performed in our study, two key considerations for Cr(VI) absorption are reduction rate and capacity of the gut lumen. Reduction of Cr(VI) occurs in the stomach due to the low pH and reducing agents (e.g., ascorbate, glutathione, NADH, sulfhydryls), whereas Cr(VI) absorption occurs in the small intestine. Humans and rats exhibit distinct feeding patterns that may influence gut reduction of Cr(VI); whereas humans are intermittent feeders, rats are continuous feeders. A previous study assessed the reductive capacity of human gastric juice pre- and post-meal consumption, reporting significant differences in pH and reductive capacities for Cr(VI) with lower pH and more reduction occurring in post-meal gastric juices than pre-meal [70]. Hence, our study interpretation may be limited due to these differences in gut Cr(VI) reduction. A previous study comparing rat and mouse gut reduction rates determined rats given 0.1 mg Cr(VI)/L in drinking water results in a stomach concentration of 0.07 mg Cr(VI)/L and estimated Cr(VI) intake per bout of drinking was 0.4% after adjusting for reducing capacity [71]. According to these data, our rats exposed to 0.1 mg Cr(VI)/L for 90 days would have a cumulative Cr(VI) intake of 84–184 mg Cr(VI) based on our water consumption data. Other factors influencing Cr(VI) intake via ingestion of contaminated drinking water include the age or sex of the individual, which can influence stomach lumen volume and pH. Studies report females have a higher pH and lower volumes of gastric juice than males, which may underlie some of the differences observed in our study [72,73,74,75,76]. However, age does not appear to influence stomach pH, though may increase risk of chronic atrophic gastritis which may reduce gastric acid output [77,78,79]. To date, no published studies have considered the influence of age or sex on Cr(VI) gut reduction in humans or rodents.

Our data clearly demonstrate brain Cr accumulation and neurotoxicity are age- and sex-specific, and follow-up studies should consider longer or lifelong exposures. Another knowledge gap remains regarding the permanence of Cr(VI) neurotoxicity, as the standard practice for drinking water Cr(VI) is removal via adsorption or reduction to Cr(III). One study reported cessation of Cr(VI) exposure reduced brain Cr levels in Japanese medaka, but these data have not been replicated in rodents [56]. This is a key consideration, as studies with other metals report toxic effects can be worse after a recovery period, and additional interventions may be necessary to attenuate such delayed toxicity [80,81,82,83].

## 5. Conclusions

From these data, we conclude Cr accumulation is age-, sex-, and brain region-specific. Our data from rats exposed to WHO and US EPA MCLs suggest drinking water Cr(VI) selectively accumulates in the hippocampus, and geriatric females accumulate the highest levels. We also observed Cr(VI) induced significant essential metal dyshomeostasis in the hippocampus, with opposite effects between sexes and when comparing between young and older rats. Because of the direct relevance to human exposures, these data suggest human populations around the world may be experiencing Cr(VI) neurotoxicity, despite their water being deemed safe for consumption. Our data suggest a need for wider and more rigorous monitoring of drinking water Cr(VI) levels to better inform populations at risk.

## Figures and Tables

**Table 1:** Cr(VI) altered essential metal levels in the hippocampus.

Sex	Age	Cr(VI) Concentration [mg/L]	Average Metal Level [ng/g] (SEM)						
			*Fe*	*Cu*	*Se*	*Mg*	*Co*	*Mn*	*Zn*
**Male**	**Young**	0	21,715 (1514)	4760 (284.6)	180.1 (22.51)	292,146 (25,170)	9.989 (1.300)	679.3 (96.14)	32,172 (4943)
		0.05	26,055 * (1495)	5466 * (177.4)	233.1 (10.03)	352,197 (28,417)	10.63 (1.137)	710.0 (18.97)	31,163 (630.8)
		0.1	22,952 (628.5)	5276 (211.5)	190.2 (7.771)	323,197 (18,114)	8.743 (0.4724)	654.4 (35.45)	30,032 (1201)
	**Middle-Aged**	0	34,197 (3200)	7823 (856.9)	290.0 (31.86)	363,367 (32,957)	12.68 (1.253)	828.7 (79.61)	57,419 (6454)
		0.05	30,202 (3967)	6750 (944.1)	230.2 (26.58)	303,851 * (39,843)	10.98 (1.812)	709.3 (102.6)	48,197 * (7121)
		0.1	16,727 * (2648)	3797 (422.5)	131.3 * (19.20)	174,813 (24,294)	5.879 * (0.8321)	407.3 * (48.68)	25,818 (3696)
	**Geriatric**	0	38,021 (2064)	12,575 (788.8)	311.9 (18.50)	383,070 (15,868)	15.16 (0.8963)	930.7 (52.42)	55,825 (2204)
		0.05	42,380 (2909)	11,845 (1,013)	252.1 (23.19)	384,404 (8595)	16.08 (1.046)	945.6 (27.73)	70,044 (8375)
		0.1	29,203 (3462)	8241 * (1254)	216.7 * (23.99)	280,733 * (35,762)	10.71 * (1.226)	640.0 * (76.23)	47,323 (4989)
**Female**	**Young**	0	29,232 (1050)	6364 (350)	196.8 (7.007)	366,401 (22,277)	11.86 (1.866)	5904 (3294)	29,338 (1030)
		0.05	28,590 * (2782)	5789 (183.5)	199.7 (11.78)	348,173 (18,855)	10.84 (0.8427)	2908 (2034)	28,868 (924.9)
		0.1	25,613 (1009)	5796 (243.8)	186.6 (12.43)	297,391 * (10,835)	15.16 (3.675)	904.8 (117.7)	26,531 (957.1)
	**Middle-Aged**	0	27,454 (4005)	6848 (1132)	193.9 (31.76)	263,149 (40,214)	9.186 (1.749)	659.4 (102.2)	39,367 (7662)
		0.05	31,179 (4310)	7187 (1002)	241.9 (23.25)	300,150 (42,064)	9.608 (1.240)	785.8 (107.4)	44,550 (6675)
		0.1	37,900 (3839)	9592 (1032)	295.5 (37.63)	386,239 * (40,187)	12.75 (1.148)	955.9 (99.93)	56,266 (6015)
	**Geriatric**	0	21,500 (4491)	5950 (797.9)	148.7 (24.74)	182,862 (25,163)	9.692 (1.436)	527.2 (83.90)	25,947 (3037)
		0.05	19,890 (2698)	6452 (707.6)	142.5 * (27.50)	181,910 (22,586)	11.45 (1.509)	508.2 (65.72)	28,143 (2979)
		0.1	38,625 * (1262)	11,807 * (299.7)	270.2 (13.24)	361,095 * (9246)	21.64 * (1.785)	936.5 * (48.48)	54,011 * (5084)

Raw data from Figure 4 are shown here. * *p* < 0.05.

## Data Availability

The data that support the findings of this study are available from the corresponding author upon reasonable request.

## Supplementary Materials

The following supporting information can be downloaded at: https://www.mdpi.com/article/10.3390/toxics12100722/s1, Figure S1: Cr Concentration in drinking water. Samples were collected weekly from the tap water and the drinking water given to rats each week to validate the exposure regimen. Data represent means ± SEMs. Note: All tap/control water samples were below the limit of detection; hence, they were set to half this limit (0.011 mg/L).
