# Supplementary material for: Chromium Selectively Accumulates in the Rat Hippocampus after 90 Days of Exposure to Cr(VI) in Drinking Water and Induces Age- and Sex-Dependent Metal Dyshomeostasis
Source: Toxics. 2024 Oct 3;12(10):722. doi: 10.3390/toxics12100722 (PMC11510846; doi:10.3390/toxics12100722)
Supplement: Supplementary file 1 [file toxics-12-00722-s001.zip › toxics-3180101-supplementary.pdf]

toxics

Supplementary Materials: Chromium Selectively Accumulates in the Rat Hippocampus after 90 Days of Exposure to Drinking Water and Induces Age- and Sex-Dependent Metal Dyshomeostasis

Samuel T. Vielee, William J. Buchanan, Spencer H. Roof, Rehan Kahloon, Ellie Evans, Jessica Isibor, Maitri Patel, Idoia Meaza, Haiyan Lu, Aggie Williams, J. Calvin Kouokam, Sandra S. Wise, Luping Guo, Rachel M. Wise, Jamie L. Wise, Lu Cai, Jun Cai and John P. Wise Jr.

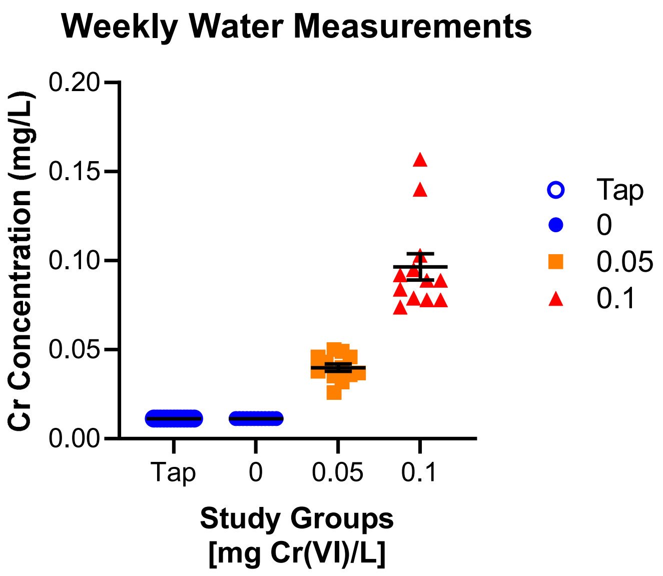

Figure S1. Cr Concentration in drinking water. Samples were collected weekly from the tap water and the drinking water given to rats each week to validate the exposure regimen. Data represent means ± SEMs. Note: All tap/control water samples were below the limit of detection; hence, they were set to half this limit (0.011 mg/L).
